# RETRACTION: Effect of Hydrogen Sulphide on Inflammatory Factors of the Mitochondria After Limb Ischaemia‐Reperfusion Injury in Rats

**DOI:** 10.1111/iwj.70632

**Published:** 2025-04-23

## Abstract

**RETRACTION:** J. Fu, X.H. Cheng, and L. Zhang, “Effect of Hydrogen Sulphide on Inflammatory Factors of the Mitochondria After Limb Ischaemia‐Reperfusion Injury in Rats,” International Wound Journal 16, no. 3 (2019): 595–600, 10.1111/iwj.13068.30693651 PMC7948542

The above article, published online on 28 January 2019, in Wiley Online Library (http://onlinelibrary.wiley.com/), has been retracted by agreement between the journal Editor in Chief, Professor Keith Harding; and John Wiley & Sons Ltd. Following an investigation by the publisher, all parties have concluded that this article was accepted solely on the basis of a compromised peer review process. In addition, further investigation by the publisher found that the authors provided incomplete ethical approval information for animal experiments performed in the study. The editors have therefore decided to retract the article. The authors did not respond to our notice regarding the retraction.

**RETRACTION:** J. Fu, X.H. Cheng, and L. Zhang, “Effect of Hydrogen Sulphide on Inflammatory Factors of the Mitochondria After Limb Ischaemia‐Reperfusion Injury in Rats,” International Wound Journal 16, no. 3 (2019): 595–600, 10.1111/iwj.13068.30693651 PMC7948542

The above article, published online on 28 January 2019, in Wiley Online Library (http://onlinelibrary.wiley.com/), has been retracted by agreement between the journal Editor in Chief, Professor Keith Harding; and John Wiley & Sons Ltd. Following an investigation by the publisher, all parties have concluded that this article was accepted solely on the basis of a compromised peer review process. In addition, further investigation by the publisher found that the authors provided incomplete ethical approval information for animal experiments performed in the study. The editors have therefore decided to retract the article. The authors did not respond to our notice regarding the retraction.

